# Editorial: Pushing boundaries in EHR implementation and innovation through advanced digital health technologies

**DOI:** 10.3389/fmed.2026.1887713

**Published:** 2026-06-29

**Authors:** Gunnar Piho, Evgeniy Krastev, Laura-Maria Peltonen, Maria Hägglund, Peeter Ross

**Affiliations:** 1 Tallinn University of Technology, Tallinn, Estonia; 2 Sofia University, Sofia, Bulgaria; 3 University of Eastern Finland, Kuopio, Finland; 4 Uppsala University, Uppsala, Sweden

**Keywords:** artificial intelligence, digital health, EHDs, electronic health records, interoperability, secondary use of health data

## Introduction and rationale

Electronic health records (EHRs) have become a central component of modern healthcare, enabling data-driven decision-making, continuity of care, and a patient-centered approach (1). At the same time, these systems still have multiple structural, technical, and organizational limitations (2). These include data fragmentation, heterogeneous data formats, limited interoperability, and legal and administrative barriers, particularly in cross-border data exchange (3–5).

At the European level, these challenges are being addressed through the European Health Data Space (EHDS) initiative, which aims to enable secure, standardized, and reusable health data flows for both primary and secondary use (de la Cruz et al.). The EHDS introduces new opportunities, including the adoption of harmonized data exchange formats, such as the European Electronic Health Record Exchange Format (EEHRxF), and the adaptation of national health information systems (Murgia et al.; da Silva Carvalho et al.).

Parallel to this, new digital health technologies are rapidly evolving, including artificial intelligence, wearable devices, automated decision support, and decentralized architectures. These developments create new opportunities for personalized, preventive, and data-driven healthcare, while also introducing new requirements for existing EHR systems [Ambalavanan et al.(a); Kumar et al.; Sarraf and Ghasempour]. Furthermore, ensuring that secondary data use is conducted with data protection, trust, and regulatory compliance has become increasingly critical (de la Cruz et al.; Vovk et al.).

This Research Topic brings together contributions that address these challenges and opportunities from multiple perspectives and examines how EHR systems can evolve to support interoperability, secure data use, clinical effectiveness, and patient-centered healthcare.

## Theme and scope of the Research Topic

This Research Topic is grounded in the aim of the Research Topic to address challenges and opportunities in the implementation and evolution of EHR systems within increasingly complex digital health ecosystems. Although EHR systems have improved data accessibility and continuity of care, their full potential remains constrained by fragmented infrastructures, heterogeneous data formats, regulatory heterogeneity, and insufficient interoperability both within and across systems, including in cross-border contexts.

A central theme of the Research Topic is interoperability at both the syntactic and the semantic level. The coexistence of diverse data models, standards, and system architectures continues to hinder effective data exchange and reuse. Addressing these limitations requires the broader adoption of standards such as HL7 FHIR, as well as reliable methods for data transformation and harmonization across heterogeneous environments [Murgia et al.; Beyer et al.; Ambalavanan et al.(a)]. Policy-driven initiatives, most notably the EHDS and the EEHRxF, introduce additional technical, interoperability, and organizational challenges that must be addressed (da Silva Carvalho et al.).

A second major focus is the secondary use of health data, which is increasingly recognized as important for research, innovation, public health, and evidence-based policymaking. Despite its considerable potential, secondary data use remains constrained by privacy requirements, data quality issues, complex governance processes, and the practical challenges associated with de-identification and risk assessment [Ambalavanan et al.(b); de la Cruz et al.; Vovk et al.]. These challenges highlight the need for solutions that balance data accessibility with the protection of individual rights and compliance with regulatory frameworks.

The Research Topic also explores the integration of emerging digital health technologies into EHR ecosystems. Rapid developments in artificial intelligence, including natural language processing, clinical decision support, and generative models, are transforming the way health data are processed and utilized. However, these developments introduce challenges related to workflow integration, usability, and their impact on healthcare professionals, particularly in terms of cognitive workload and burnout [Ambalavanan et al.(a); Sarraf and Ghasempour]. In addition, decentralized architectures provide alternative approaches to data management, emphasizing patient control, transparency, and security, while raising important questions regarding scalability, governance, and practical implementation (Kumar et al.).

Finally, the Research Topic considers the broader legal, ethical, and societal context of EHR use. Issues such as citizen rights, trust in data reuse, informed consent (including opt-out mechanisms), and public engagement in health data governance are essential for ensuring the legitimacy and sustainability of digital health systems (de la Cruz et al.). These considerations are particularly significant in the context of large-scale initiatives, such as the EHDS, where the success of technical and regulatory frameworks depends on public trust and meaningful participation.

Overall, the Research Topic shows the need for a comprehensive and interdisciplinary approach to EHR innovation that addresses existing limitations and supports the integration of emerging technologies and governance models aimed at enabling more efficient, secure, patient-centered, and sustainable digital healthcare systems. At the same time, the contributions in this Research Topic provide concrete examples of how such integration is already being operationalized through specific technical solutions, policy frameworks, and implementation strategies.

## Overview of the selected articles

The articles included in this Research Topic address the development and implementation of EHR systems from multiple perspectives, offering both conceptual insights and practical solutions.

Ambalavanan et al.(a) examine ontologies as a semantic bridge between artificial intelligence and healthcare. Their work shows that semantic modeling is needed to make clinical, genomic, and phenotypic data from diverse sources interpretable by both machines and humans, supporting interoperability, decision support, and personalized medicine. The article synthesizes and systematizes the role of ontologies in applying artificial intelligence in health care and identifies the need for adaptive, privacy-aware, and patient-centered ontologies.

A related systematic review by the same authors highlights that the development of robust digital health data ecosystems requires the simultaneous resolution of technical, organizational, and regulatory barriers [Ambalavanan et al.(b)]. The article contributes a systematic review and thematic synthesis of healthcare data integration, identifying key challenges and consolidating existing strategies across interoperability, patient-centered care, and genomic data integration to provide an evidence-based foundation for developing scalable and secure digital health ecosystems.

Sarraf and Ghasempour analyze, through a systematic review, the impact of artificial intelligence on EHR-related burnout among healthcare professionals. Their findings suggest that AI-based solutions, such as ambient documentation tools, natural language processing applications, and decision support systems, can reduce documentation burden and improve workflow efficiency, although further methodological rigor is needed to strengthen the evidence base. The article contributes a systematic synthesis of evidence showing that AI integrated into EHR systems can reduce EHR-related burnout among healthcare professionals, especially by easing documentation burden and inbox management, while also identifying the main limitations, risks, and research gaps of these interventions.

Murgia et al. investigate national health data ecosystems in the context of the EHDS, highlighting challenges related to fragmentation, decentralization, and the inconsistent implementation of standards. Using the Italian case as an example, the study demonstrates how regional autonomy can hinder the adoption of unified standards and impede seamless data exchange at both national and European levels. The article analyzes how national health data ecosystems, using Italy as a case study, face organizational, technical, legal, and interoperability challenges in aligning with the European Health Data Space (EHDS). It also discusses the strategies, standards, and initiatives needed to enable secure, interoperable, and privacy-compliant health data sharing across decentralized systems.

Beyer et al. focus on technical solutions by developing an open-source compiler for transforming health data across formats. Their work demonstrates that data transformation is a critical component of achieving interoperability and that practical tools must be efficient, transparent, and portable to support practical implementation. The article contributes the design and implementation of an open-source compiler (MaLaC-HD) that translates FHIR mapping language (FML) into transparent and portable executable code, improving the speed and practicality of health data transformations.

Vovk et al. address the challenges of secondary data use by proposing the WiseSpace solution, which provides mechanisms for the de-identification, risk assessment, and controlled re-identification of health data. Their study highlights the need to balance privacy protection and data usability so that health data can be used for research and other societal purposes while still meeting regulatory requirements. The article contributes the design and evaluation of WiseSpace, a healthcare-specific de-identification tool that enables the secure, regulation-compliant secondary use of electronic health record data through automated identifier detection, anonymization, and re-identification risk assessment accessible to non-technical domain experts.

da Silva Carvalho et al. examine policy-level challenges related to the implementation of the EEHRxF across Europe. Their analysis emphasizes the need for coordinated action among stakeholders, including system vendors, healthcare providers, standardization bodies, and public authorities, and offers practical recommendations. The article contributes a policy-oriented implementation framework for the European electronic health record exchange format (EEHRxF) by comparing implementation models and introducing practical mechanisms, such as a voluntary interoperability label and the “Yellow Button” concept, to accelerate interoperable, patient-centered health data exchange across Europe.

de la Cruz et al. approach the EHDS from the perspective of citizen rights and health data governance. Their study shows that the legitimacy of secondary data use depends on legal frameworks, citizens' understanding of the system, their perception of its value, and their ability to exercise meaningful control over their data. The article contributes empirical evidence from expert interviews across Europe, showing that effective implementation of the European Health Data Space requires formal citizen rights, such as opt-out mechanisms, practical mechanisms for meaningful citizen engagement, clear communication, and a clear explanation of the public benefit from health data reuse.

Kumar et al. explore decentralized digital health architectures, proposing an integrated framework that combines blockchain, artificial intelligence, and edge computing. Their approach emphasizes patient control, security, verifiable identity management, and transparent governance as key features of next-generation health information systems. The article presents a decentralized architecture (PolyMed) that integrates blockchain, artificial intelligence, edge computing, privacy-preserving identity management, and decentralized governance into a unified patient-centered health data management system.

Taken together, these articles show the current developments in EHR implementation and digital health innovation.

## Cross-cutting insights and emerging directions

The articles identify several common themes. First, data fragmentation and the inconsistent implementation of standards remain central challenges, limiting interoperability at both national and international levels. These issues are not purely technical; they also reflect differences in governance models, legal interpretations, and levels of institutional maturity (Murgia et al.; da Silva Carvalho et al.).

Second, the central role of semantic interoperability in the effective use of data becomes evident. Without a shared conceptual and terminological foundation, even advanced artificial intelligence applications and data exchange mechanisms remain limited in their effectiveness. Ontologies, standards, and controlled vocabularies are therefore fundamental components of digital health infrastructure [Ambalavanan et al.(a)].

Third, data transformation, standardization, and portable technical tools are important. Within EHR ecosystems, abstract standards alone are insufficient; practical, implementable solutions are required to connect existing systems with evolving technical and regulatory requirements (Beyer et al.; da Silva Carvalho et al.).

Fourth, health data use must balance data accessibility and usability with privacy protection. The secondary use of health data depends on reliable mechanisms for de-identification, risk assessment, and data access governance. Success is not determined solely by technical safeguards, but also by public trust, transparent communication, and clear explanation of the public benefit from data reuse (de la Cruz et al.; Vovk et al.). At the same time, these dimensions are not always aligned: increased data accessibility for research and innovation may conflict with individual control and expectations of privacy, indicating the need to balance these priorities.

Fifth, the contributions indicate that the application of artificial intelligence in EHR environments should not be limited to expanding analytical capabilities, but should address practical constraints in clinical workflows. When appropriately implemented, AI can reduce documentation burden, improve data interpretability, and support transparent decision-making, thereby improving system acceptance and the wellbeing of healthcare professionals [Ambalavanan et al.(a); Sarraf and Ghasempour]. Since only a limited number of contributions focus on AI, broader issues such as robustness, fairness, explainability, and long-term clinical impact remain only partly addressed in this Research Topic. The included contributions therefore provide concrete evidence and practical examples of AI integration in EHR systems, especially for workflow optimization and decision support, but they do not cover the full scope of AI-related challenges.

Finally, there is a visible shift toward more patient-centered and distributed architectures. Decentralized models, verifiable identity mechanisms, and enhanced patient control over data have the potential to change institution-centered EHR models (Kumar et al.). However, these approaches still require further evaluation in terms of usability, clinical relevance, and large-scale implementation. Decentralized, patient-controlled data architectures and the more centralized governance and coordination mechanisms used in large-scale initiatives such as the EHDS follow different logics. This raises important questions about how control, accountability, and efficiency should be balanced in future health data ecosystems.

## Implications for research and practice

The contributions in this Research Topic point to the need for an integrated approach that brings together technical solutions, standardization, regulation, workflow redesign, and user-centered design. EHR innovation cannot be effective if it is addressed in isolation at a single level.

From a research perspective, there is a clear need for new frameworks and methodologies capable of evaluating EHR systems in terms of interoperability, semantic quality, clinical usability, and their impact on healthcare sustainability and accessibility. Given the methodological diversity across the included studies, future research should address how different types of evidence, such as technical validation, design science, systematic reviews, and policy analysis, can be integrated to support robust and comparable conclusions. Further work is required to develop methods that support the secure reuse of health data and enable the application of artificial intelligence in ways that are trustworthy, transparent, and clinically appropriate [Ambalavanan et al.(b); Ambalavanan et al.(a); Sarraf and Ghasempour]. At the same time, the Research Topic offers existing solutions and approaches that can already inform ongoing research and implementation efforts.

In practice, this implies coordinated action among multiple stakeholders, including system developers, healthcare providers, standardization bodies, and policymakers. The implementation of initiatives such as the EEHRxF and the EHDS requires clear guidance, supporting infrastructures, realistic transition mechanisms, and practical tools that facilitate the integration of existing systems with emerging requirements (Murgia et al.; Beyer et al.; da Silva Carvalho et al.).

Equally important is the need to address issues of trust. Citizen engagement, understandable opt-out or other control mechanisms, transparent communication, and clear public benefit are all essential to ensure that the sharing and reuse of health data are socially acceptable (de la Cruz et al.). In parallel, healthcare organizations must be provided with usable solutions for de-identification and risk assessment that lower the technical barriers to compliant data sharing (Vovk et al.).

Finally, several contributions indicate that future EHR systems should increasingly adopt modular and extensible architectures capable of integrating distributed technologies and intelligent services. This requires a shift in perspective, where innovation is not treated as the addition of isolated components, but as the design and evolution of a coherent digital health ecosystem (Kumar et al.).

## Concluding remarks

The development of EHR systems has reached a stage where technological capabilities often exceed the organizational, semantic, and regulatory capacities of existing systems. The contributions in this Research Topic demonstrate that meaningful progress requires the simultaneous advancement of both technical solutions and organizational structures.

At the same time, this Research Topic illustrates that progress is shaped by shared goals as well as unresolved differences between alternative architectural, governance, and implementation approaches.

Interoperability, standardization, secure data use, trustworthy governance, and patient-centeredness emerge as key factors shaping the future of EHR systems. The EHDS provides a strong political and regulatory framework to support these objectives; however, its successful implementation depends on the availability of practical tools, coordinated collaboration among stakeholders, and the ability to align innovation with the real-world needs of healthcare systems (de la Cruz et al.; da Silva Carvalho et al.).

The Research Topic brings together work that addresses multiple related aspects of EHR innovation, including semantic modeling, artificial intelligence, secondary use of health data, policy frameworks, data transformation tools, and decentralized architectures. Together, these contributions point to a future in which EHR systems evolve from fragmented, registry-based infrastructures into more integrated, trustworthy, and intelligent digital health ecosystems [Beyer et al.; Ambalavanan et al. (a); Kumar et al.; Vovk et al.]. Importantly, the main value of this Research Topic lies in the specific knowledge, methods, and results presented in the individual articles, which together provide a basis for further research, implementation, and citation.

However, the limited coverage of certain themes and perspectives indicates the need for further research to establish a broader evidence base, particularly with respect to clinical outcomes, broader stakeholder perspectives, empirical evidence on patient outcomes, non-European contexts, and detailed analyses of nursing or allied health workflows and emerging technological risks. Yet, within this Research Topic, the included articles provide a substantive contribution to research on EHR implementation and digital health technologies.

## References

[1] Blobel B. Interoperable EHR systems-challenges, standards and solutions. Eur J. Biomed Informat. (2018) 14:10–9. doi: 10.24105/ejbi.2018.14.2.3

[2] Pedrera-Jiménez M García-Barrio N Frid S Moner D Boscá-Tomás D Lozano-Rubí R . Can OpenEHR, ISO 13606, and HL7 FHIR work together? An agnostic approach for the selection and application of electronic health record standards to the next-generation health data spaces. J Med Internet Res. (2023) 25:e48702. doi: 10.2196/48702 38153779 PMC10784985

[3] Bincoletto G. Data protection issues in cross-border interoperability of Electronic Health Record systems within the European Union. Data Policy. (2020) 2:e3. doi: 10.1017/dap.2020.2

[4] Torab-Miandoab A Samad-Soltani T Jodati A Rezaei-Hachesu P. Interoperability of heterogeneous health information systems: a systematic literature review. BMC Med Inform Decis Mak. (2023) 23:18. doi: 10.1186/s12911-023-02115-5 36694161 PMC9875417

[5] Van Scharen A Cruyt K Colon JJE De Sutter S Duerinck J Cornu P . Unlocking health data for research: legal, technical, and organisational lessons from a belgian interdisciplinary case study. J Healthc Informat Res. (2025) 10:179–208. doi: 10.1007/s41666-025-00220-w PMC12873005 41658397

